# A Teleneuropsychological Battery for Assessing Older Panamanian Adults: Protocol for a Cross-Sectional Pilot Feasibility Study

**DOI:** 10.2196/68520

**Published:** 2025-08-28

**Authors:** Luis Guillermo Santos Mejía, Diana C Oviedo, Ambar Perez-Lao, Gabrielle B Britton, Glenn Smith

**Affiliations:** 1 Instituto de Investigaciones Científicas y Servicios de Alta Tecnología Panama City Panama; 2 Universidad Católica Santa María La Antigua Panama City Panama; 3 Sistema Nacional de Investigación, SENACYT Panama City Panama; 4 University of Florida Gainesville, FL United States; 5 CEVAXIN Panama City Panama

**Keywords:** cognition, older adults, telehealth, teleneuropsychology, protocol, Hispanic population

## Abstract

**Background:**

Evidence has shown that teleneuropsychology is a crucial tool in the assessment and treatment of individuals with limited access to in-person health services. Nevertheless, studies in Latin America are scarce, and there is a pressing need for studies that test the feasibility of teleneuropsychology and contribute to the development and validation of tools and models, as well as the establishment of normative data for the diverse populations of the region.

**Objective:**

This study aims to conduct the first pilot study in Central America to assess the feasibility of a teleneuropsychology protocol and to generate normative data for a Panamanian sample.

**Methods:**

This is a cross-sectional, descriptive, community-based study derived from the Development and Validation of Teleneuropsychology Normative Data for Older Adults in Florida (FLOAT) study. The study’s final sample will include 150 participants (aged ≥50 years). Participants will undergo initial screening to determine their eligibility and will be included if they have a basic understanding of and access to technological devices such as telephones, tablets, or computers; are free of cognitive impairment or serious health conditions; and provide informed consent. Participants will complete 2 questionnaires to determine if they can continue in the study. Participants who are eligible will be assessed with a teleneuropsychology cognitive battery and will answer questionnaires via a REDCap (Research Electronic Data Capture) link.

**Results:**

Feasibility will be assessed based on an analysis of the tools, funding, expertise, and resources needed to conduct neuropsychological assessments. So far, the study has proven feasible as researchers have been able to obtain a REDCap license and all other material necessary to carry out assessments. As of November 11, 2024, a total of 67 participants aged ≥50 (mean 62.2, SD 7.6) years had been assessed with questionnaires and a complete cognitive test battery. Sociodemographic and clinical characteristics show that participants on average had 16.7 (SD 1.9) years of formal education. A satisfaction questionnaire revealed that most participants were satisfied (22/67, 33%) or very satisfied (39/67, 58%) with the teleneuropsychological assessment, and most participants (58/67, 86%) would recommend this study to others. We expect to assess a similar number of women and men as well as different age groups with the teleneuropsychology battery. We plan to generate normative data for Panamanian adults aged >50 years.

**Conclusions:**

This study may point to the need to use assessment methods that are convenient and efficient for older adult populations and that can be used as substitutes for in-person evaluations. Although it is a viable approach, expanding the sample size is essential to generate reliable normative data. This is an unprecedented area of research in the country and the Central American region.

**International Registered Report Identifier (IRRID):**

DERR1-10.2196/68520

## Introduction

### Background

Teleneuropsychology refers to the use of audiovisual technologies for remote neuropsychological assessments and interventions [1]. Specialty psychological services, namely, neuropsychological assessments, seek to integrate digital tools into conventional practice, combining traditional in-person measures for rich clinical observations with novel digital or remote assessments that allow for more precise quantification of behavioral patterns [2]. Teleneuropsychology requires the use of various tests and tools to evaluate a person’s current cognitive and emotional status. This service usually requires patients to visit assessment sites and undergo clinical interviews and neuropsychological testing. Nevertheless, health emergencies such as the COVID-19 pandemic hindered in-person visits, prompting mental health professionals to adopt remote evaluations or teleneuropsychological assessments as an alternate method. The Inter Organizational Practice Committee created new guidelines for the practice of teleneuropsychology during the pandemic [3,4] to aid with some of the difficulties that are common in teleneuropsychology, such as uncontrolled conditions of in-home assessments [5]. In Latin America, recommendations on the use of teleneuropsychology emerged during the pandemic, with a lack of normative data for various countries [3]. However, teleneuropsychology did not arise solely as a response to health emergencies. Before the COVID-19 pandemic, guidelines for remote testing had been created by the American Psychological Association and Joint Task Force for the Development of Teleneuropsychology Guidelines for Psychologists [6]. These guidelines were developed to address ethical and methodological challenges associated with internet-based assessments, such as limited confidentiality and security risks when using the internet. One key recommendation was to incorporate ethical limitations into the informed consent process, providing participants with a clear and thorough explanation of the unique aspects of teleneuropsychology assessments [7]. Moreover, historically, studies have explored the validity and feasibility of remote care, initially through telephone and later through videoconferences.

### Teleneuropsychology: How It Came to Be and Its Development Before the COVID-19 Pandemic

Teleneuropsychology’s origins date back to the 1980s, when telephones were used for patient follow-up and cognitive health assessments. By then, widespread telephone use in US households facilitated telephonic data collection, particularly in primary care for consultations and surveys. In the 1990s, telephones were used for follow-ups with patients and screenings to detect dementia in older adults [8] as they function as a cost-effective alternative to in-person surveys [9]. In this period, there was an initial exploration of the feasibility of videoconferencing for cognitive evaluations in older adults [10,11] and an ongoing development and validation of remote tests [12-21]. Telephone screening tools, such as the modified Telephone Interview for Cognitive Status (TICS-M) and the Mini-Mental Status Examination, offer benefits such as rapid administration and cost-effectiveness, making them suitable for large-scale epidemiological studies [22]. These tools can also help minimize dropout rates in longitudinal studies and overcome geographical barriers [23]. Older adults, who may have mobility issues or reduced motivation, often respond well to telephone assessments, enabling “cognitive triage” and follow-ups in hard-to-reach populations [24]. However, the effectiveness of telephone assessments largely depends on the design of the screening instruments [25]. In this context, videoconferencing presents several limitations, including the inability to assess praxis or visuospatial abilities [26], limited use of visual stimuli, and the examiner’s restricted ability to directly observe behavior in an uncontrolled environment [27]. In addition, communication challenges arising from hearing loss or cognitive decline further complicate the assessment process [28].

Since the early 21st century, research has explored the feasibility of teleneuropsychological assessments. Studies have shown no significant performance differences between videoconference and face-to-face assessments, demonstrating its feasibility and potential utility [27-31]. Furthermore, teleneuropsychology has not hindered clinical intervention or rapport building [29,30]. Diagnostic agreement between on-site and videoconference screenings for neurocognitive disorders has also been confirmed [32,33]. Likewise, teleneuropsychological assessments have shown to be feasible in different clinical settings, such as assessment of survivors of stroke [34]. Acceptability [35] and satisfaction [36] with teleneuropsychology have been documented, including assessments designed for use in Latin populations [37] and other underrepresented populations [38].

### State of Teleneuropsychology During COVID-19: Uses, Validation, and Research

Before the pandemic, a survey revealed that approximately one-fourth of professionals, primarily from the United States, used teleneuropsychology for clinical interviews and interventions [39]. This highlighted the underuse of teleneuropsychology, which was largely due to lack of reimbursement from Medicare and private insurance [40]. Challenges such as limited access to technology, inability to perform hands-on assessments, and reduced behavioral observation opportunities also hindered its adoption [41]. However, lockdowns increased the need for recommendations and research, leading to greater use of teleneuropsychology among practitioners [5,42-44]. Research during the COVID-19 pandemic underscored the necessity for further investigation into teleneuropsychology and its limitations. Individuals with lower incomes often lacked access to necessary technology [45,46]. In addition, participants’ computer literacy skills were frequently inadequate, and there was a scarcity of research on teleneuropsychology’s application in home health care [33,47-49].

Research in teleneuropsychology, especially concerning culturally diverse populations, has progressed following the declaration of the end of the COVID-19 pandemic [50-52]. Notably, neuropsychologists have reported significant use of this work method [53]. In addition, new tools such as the Tele-Global Examination of Mental State [54] and Tele Executive Function for children [55] have shown promising results.

Studies on validity have shown that standardized cognitive tests used in teleneuropsychology retain their psychometric properties and yield results comparable to in-person administration [56]. Similarly, research shows that teleneuropsychology assessments are reliable, as they often show stable results when repeated over time, indicating reliability in tracking cognitive performance [57]. In addition, remote assessments allow for broader participation while maintaining standardized administration protocols through platforms such as Zoom (Zoom Video Communications), REDCap (Research Electronic Data Capture; Vanderbilt University), and other secure testing software [5].

### Teleneuropsychology in Latin America: Research and Practice

Until 2021, no studies on teleneuropsychology had been reported in Latin America [41]. However, from 2021 to date, 8 studies have been documented regarding the use of teleneuropsychology in 4 countries in the region. Most of the studies are descriptive, and only 1 is a randomized controlled trial. In 38% of the studies, a satisfaction survey was included. In half of the studies, teleneuropsychology was used to evaluate participants, and in 1 study, telerehabilitation was performed. Results indicated good acceptance of teleneuropsychology as an assessment or rehabilitation modality by both participants and professionals [49,58,59]. Key benefits included reducing the stress of traveling to evaluation sites, increased accessibility to remote areas, increased comfort of home assessments [45,60], continued access during social distancing, and reduced COVID-19 risk [59]. Moreover, challenges noted by practitioners included patients’ unfamiliarity with technology; lack of environmental control; and insufficient access to technological resources, including internet connectivity [46].

These studies represent a significant initial progress in Latin America, revealing insights into the experiences of neuropsychology professionals in the region. Although Mexico and Argentina are middle-income countries, they face findings and challenges similar to those observed in high-income nations. Moreover, proposals for clinically applicable models are beginning to emerge in Latin America [61]. However, normative data in the region are scarce, a phenomenon not unique to the teleneuropsychology, and precedes the COVID-19 pandemic as a recurrent issue in Latin America clinical practice [62]. There is a pressing need for studies that test the feasibility of teleneuropsychology and contribute to the development of tools, models, and validations and the establishment of normative data for the diverse populations of the region.

The objective of this study is to conduct the first pilot study in Central America to assess the feasibility of a teleneuropsychology protocol and to generate normative data for a Panamanian sample. The specific objectives of the study are to (1) administer a teleneuropsychological assessment to a Panamanian sample of participants identified as having a low risk of developing dementia, (2) generate normative teleneuropsychological data from a Latin American sample in Panama to compare with a Hispanic sample in the United States, (3) compare performance on teleneuropsychological tests across different gender and age groups, and (4) assess the sample’s level of satisfaction with the administration of the teleneuropsychological evaluation battery using a standardized scale.

## Methods

### Participants

**Figure 1:**
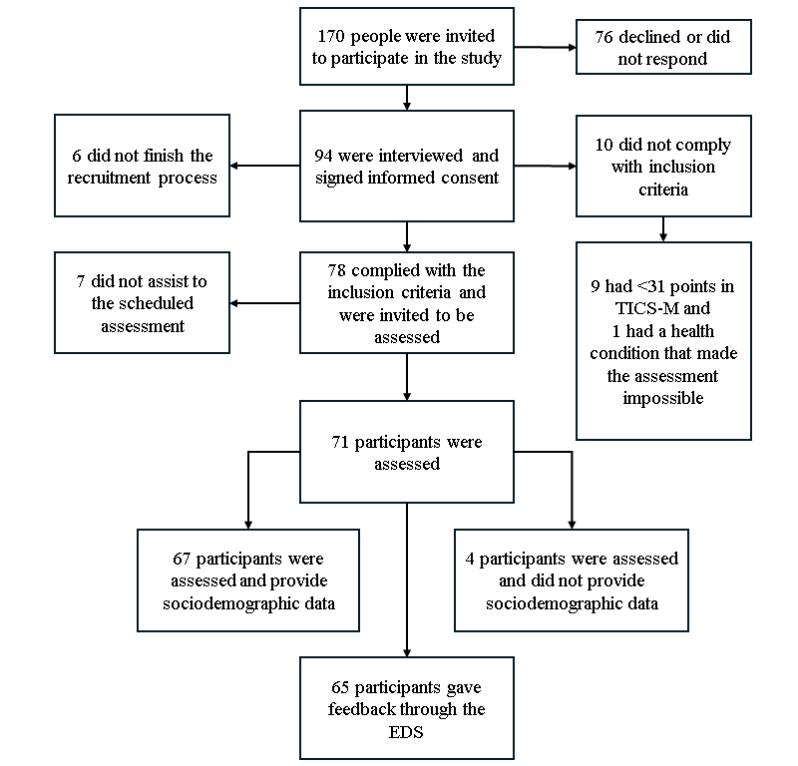
Flowchart of participants included and excluded in the study. EDS: Escala de Satisfacción; TICS-M: modified Telephone Interview for Cognitive Status.

The protocol of this study is adapted from the Development and Validation of Teleneuropsychology Normative Data for Older Adults in Florida (FLOAT) [63], a study being conducted at the University of Florida with the aim of validating a battery in a healthy sample of older adults with low risk of developing Alzheimer disease and related dementias (ADRD). This study is cross-sectional, descriptive, and community based. As of November 11, 2024, we have recruited 67 (44%) out of 150 participants, all aged ≥50 years and residing in Panama. Details of participants invited to participate, recruited, and assessed are shown in Figure 1.

### Eligibility Criteria

Participant eligibility criteria are presented in Textbox 1.

**Participant eligibility criteria.**

**Inclusion criteria**

- Are aged ≥50 years at the time of enrollment
- Have a basic understanding and control of technological devices
- Have access to a phone, tablet, or computer
- Have signed informed consent
- Are available to assist to all the program visits included in the study
- Has no cognitive impairment at the time of enrollment
- Has no conditions that limit or prevent them from completing the neuropsychological evaluation (ie, blindness, analphabetism, and severe physical limitations)

**Exclusion criteria**

- A diagnosis of Alzheimer disease and related dementias or other neurological disorders that affect cognitive functioning
- A history or diagnosis of a severe psychiatric disease (ie, schizophrenia)
- Evidence of cognitive impairment (modified Telephone Interview for Cognitive Status <31)
- No access to internet
- Lack of access to a smartphone with a webcam for videoconference
- Ongoing participation in another clinical study or the intention to participate in another study

### Recruitment

**Figure 2:**
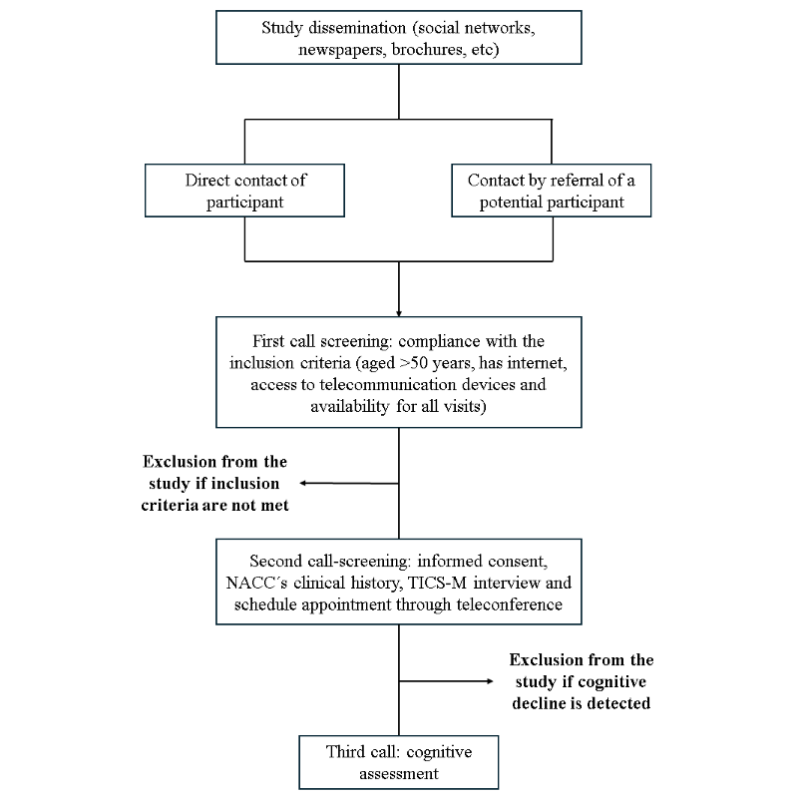
Flowchart of the recruitment process. TICS-M: modified Telephone Interview for Cognitive Status; NACC: National Alzheimer’s Coordinating Center.

Potential participants are recruited from the community in Panama through social media advertisements, flyers in community centers, and newspaper announcements. Individuals living outside the city are also encouraged to participate due to the availability of remote testing. The research team, which includes the principal investigator, research assistants, and trained psychology students, supervised by senior scientists, assist in recruitment and data collection. Participant recruitment process is displayed in Figure 2.

### Procedure

Participants undergo an initial screening to determine their eligibility. Those who meet the inclusion criteria and agree to participate will provide telephone-informed consent. Following this, participants complete 2 questionnaires: an adapted Spanish version of the modified TICS-M and the National Alzheimer’s Coordinating Center (NACC) Health Questionnaire. These assessments help identify any underlying neurological diseases or uncontrolled medical conditions that may impact cognition, such as depression or bipolar disorder. The screening aims to (1) confirm that the participant does not have cognitive impairment (eg, mild cognitive impairment or dementia) and (2) ensure that they have the cognitive capacity to provide informed consent. Participants scoring ≤31 points in TICS-M, indicating mild cognitive impairment or dementia, are excluded from the study. Those who do not meet the eligibility criteria are thanked for their time, and the assessment is discontinued.

Eligible participants receive a link to complete the questionnaires and surveys via email using the REDCap platform, an online platform designed for data collection and storage [64]. This process takes approximately 30 minutes. Later, a member of the research team schedules a 2-hour block for the teleneuropsychological assessment with ≥1 breaks if requested by the participants. A team member assists participants in setting up the videoconferencing technology. Before beginning the study, research staff reviews the informed consent document with the participant and addresses any questions.

REDCap is used to capture and store participant data, while Zoom facilitates the administration of neurocognitive tests. The screen-sharing feature is used to display test stimuli. For tasks requiring a scored motor component (eg, figure drawing), participants are asked to hold their drawings up to the camera, and a screenshot is taken for later scoring. These screenshots do not include any identifying information about the participants. Captured images are stored in the participant’s REDCap dataset for future analysis. During the assessment, 2 computers are used: a laptop and a touchscreen device, both of which project visual stimuli via the screen-sharing function. These stimuli are displayed in PowerPoint, and data collection is conducted in REDCap throughout the assessment.

### Measures

#### Overview

Following the FLOAT protocol, this study uses an open-access battery in Spanish that is part of the Uniform Data Set version 3 (UDSv3), comprising longitudinal data collected since 2005 at the National Institute of Aging funded Alzheimer’s Disease Research Centers (NACC, 2023). All measures in this study have been adapted for use with REDCap. In addition, several questionnaires, including the Health History, Functional Activity Index, Geriatric Depression Scale (GDS), and Demographic Information, have been modified to be completed directly by participants.

The evaluation process lasts approximately 3 hours, which includes 30 minutes for informed consent and screening measures, 30 minutes for participant-completed questionnaires via the REDCap platform, and approximately 2 hours for teleneuropsychological testing.

#### Screening Measures

The TICS-M [65] is administered in a Spanish version to screen for mild cognitive impairment. TICS-M measures domains such as orientation, verbal memory, attention, working memory, or abstraction and has no visual stimuli. Cognitive impairment is established at ≤31 points. This cutoff was used to determine which individuals could participate. Certain items have been modified to better suit the Panamanian context, such as replacing the question “Who is the president of the United States?” with “Who is the president of Panama?” In addition, the NACC Health History [66] measure is used to evaluate the participant’s general health history, helping to identify potential neurodegenerative diseases or other risk factors that may disqualify them from participation in the research.

#### Questionnaires

These questionnaires were obtained in the Spanish versions. Participants fill out these questionnaires online using a REDCap link provided by the study team.

The NACC-Demographics questionnaire [66] has been adapted to better suit the Panamanian population by modifying relevant questions and removing those that are not applicable, such as enquiries about the last digits of the zip code or the Alzheimer’s Disease Research Center. To assess symptoms of depression, the Spanish version of the GDS [67] is included, consisting of 30 yes or no questions completed via a REDCap form. If a participant scores >20 on the GDS, the study team contacts them to enquire about suicidal ideation. The Functional Activity Index [66] is used to evaluate an individual’s ability to perform daily activities, which may decline due to neurodegenerative processes. In addition, the Teleneuropsychological Assessment Satisfaction Scale provides participants with an opportunity to give feedback on various aspects of the assessment, including satisfaction, the use of Zoom and REDCap, instructions, advantages, difficulties, recommendations, and overall time.

#### Teleneuropsychological Tests

A variety of cognitive assessments are used to evaluate different cognitive domains. The Montreal Cognitive Assessment [68] is administered in an adapted Spanish version, serving as a cognitive screening tool that measures visuospatial skills, naming, verbal memory, working memory, attention, language, abstraction, and orientation. Verbal memory is further assessed using the Craft Story test [69], in which participants listen to a semantically related story and recall it both immediately and after a 20-minute interval, aiming to maintain the original phrasing. Similarly, the Rey Verbal Learning Test [70] requires participants to learn a list of 15 words through repeated exposure. After 5 trials, an interference list is introduced, and after a 20- to 30-minute delay, participants attempt to recall the original list, evaluating both immediate and delayed memory recall, as well as word recognition.

Visual memory and visuoconstruction skills are measured through the Benson Complex Figure test [71], where participants first copy a complex figure and then recall and recognize it after 10 minutes. Naming ability is assessed using the Multilingual Naming Test [72], in which participants observe and name figures, receiving semantic or phonemic cues if needed. The Digit Span Test [73] evaluates attention and working memory by having participants repeat a sequence of numbers in both forward and backward order, with increasing difficulty. Verbal phonological fluency [74] is assessed through a task where participants must generate as many words as possible starting with a given letter within a set time (excluding names, numbers, or repeated words). In the semantic fluency task [74], participants generate words belonging to a specific category, such as animals, vegetables, or fruits.

Executive function is measured using the Wisconsin Card Sorting Test-64 [75], a computer-based assessment where participants match cards according to rules they must infer, with alternative verbal response options available if they cannot control the screen. In addition, the Oral Trail Making Test is also used to measure executive functions. Divided in part A and part B, participants are first asked to count from 1 to 25 as fast as they can (part A). Later, they are requested to alternate between numbers and letters from 1 to 13 (part B). On both occasions, the time to finish, errors, and correct answers are recorded [76].

The Boston Naming Test [77], using a 15-item version from the Mayo Older Americans Normative Studies, presents participants with images to name, offering semantic or phonemic cues if necessary. Speeded information processing is evaluated using the oral version of the Symbol Digit Modality Test [78], in which a PowerPoint slide is displayed while responses are recorded in REDCap. Finally, premorbid intelligence is assessed with the Word Accentuation Test Chicago [79], where participants read a list of words and attempt to place the correct accentuation.

### Statistical Analyses

Neuropsychological test results will be analyzed using multivariate statistical methods. We will perform initial data analyses with chi-square and ANOVA tests to observe distribution across age groups or other groups of interest. We will obtain raw scores of each test as well as composites of cognitive domains to conduct further analyses per test and per cognitive domain. Comparisons will be made regarding sex and age groups. Student *t* test will be applied to examine differences in performance between sexes and within age groups (the sample will be divided in 3 groups: aged 51-59 years, 60-69 years, and >70 years). Multivariable regression analyses will be performed to assess linear relationships between age, years of education, or other (ie, income) variables and cognition. Once we have assessed and ruled out potential interaction effects influencing participant performance, we will proceed with an in-depth analysis of normative data stratified by age, sex, and education level. Test scores will be standardized into *z* scores based on the mean and SD of the respective group, and these *z* scores will be subsequently converted into normative scores for each assessment. Cases with incomplete data will be excluded from analyses via listwise deletion.

### Ethical Considerations

The study protocol was approved by the Research Bioethics Committee of Universidad Santa María La Antigua (CBI-USMA; 2022-P004). Informed consent is read to participants via phone call. After verbal confirmation of acceptance to participate in the study, participants get a Google Forms link were they formally accept to be part of the study and attach an image of their personal sign. Once they finish filling the forms, they are assigned a unique code.

All data collected will be saved in a password-protected database. Only authorized research personnel will have access to this database. If a participant chooses to withdraw from the study, the data collected before their withdrawal will still be included in the analysis. Data collected on REDCap are identified with a specific number to protect the identity of the participant.

Some estimated harms that could be caused by this type of evaluations include fatigue, stress, or anxiety while either responding to the questionnaires or performing the neuropsychological tests. However, we expect minimal harm to our participants. If the participant needs it, the evaluation is rescheduled. Participants are informed that they are free to withdraw from the research study at any time. As exclusion criteria indicates eligibility for participating is based on participants not having a diagnosis of ADRD or other neurological disorders that affect cognitive functioning, all participants contacted and included in the study are cognitively healthy. Nevertheless, if a participant with a diagnosis of ADRD contacts the research team, researchers will refer them to a neurologist.

### Data Management

All data, including consent, will be added to a REDCap database that has all the measures obtained in this study. To ensure high-data quality collection, scoring of the tests is reviewed by 2 different evaluators at the end of the testing. This is done to improve interrater reliability in test scoring. All evaluators have been trained using the same tools and in a standardized environment to minimize variability in score interpretation. Test scoring instructions are followed precisely.

### Dissemination Policy

Results from this research will be published, shared, and presented at national and international conferences, science events, and high-impact journals. At the end of the study, participants will be given a summary of their results that will give them an insight of their cognitive status. The report will be divided in different cognitive domains, including “below average,” “average,” and “above average” to identify the participant’s cognitive level when compared with other peers of the same age range, sex, and education.

## Results

### Preliminary General Characteristics of the Sample

The demographic and clinical characteristics of the sample are summarized in Tables 1 and 2. Participants were mostly female (53/67, 79%) and were on average aged 62.2 (SD 7.6) years. Participants on average had 16.7 (SD 1.9) years of formal education. Most participants have a partner (44/67, 66%). Participants reported few cardiovascular, cerebrovascular, and psychiatric illnesses. Nevertheless, the most common chronic illness is hypertension (22/67, 33%), followed by hypercholesterolemia (19/67, 28%). Most participants identify as White (31/67, 46%) or other (20/67, 30%). Participants showed a high degree of functional independence in performing basic and instrumental activities of daily living. On average, participants had 1.5 (SD 1.8) symptoms of depression.

**Table 1:** Protocol measures for teleneuropsychology assessment.

Measure			Description
**Screening measures**			
	TICS-M^a^	Measures domains such as orientation, verbal memory, attention, working memory, or abstraction and has no visual stimuli; cognitive impairment is established at ≤31 points	
	NACC^b^ Health History	Evaluates general health history, identifying potential neurodegenerative diseases or other risk factors that may disqualify participants in the research	
**Questionnaires**			
	NACC-Demographics Questionnaire	Addresses topics of age, sex, education, independence, type of housing, and civil status, among others.	
	GDS^c^	Consists of 30 yes or no questions completed via a REDCap^d^ form; if a participant scores >20 on the GDS, the study team enquires about suicidal ideation	
	The Functional Activity Index	Evaluates an individual’s ability to perform daily activities	
**Teleneuropsychological tests**			
	Montreal Cognitive Assessment	Cognitive screening tool that measures visuospatial skills, naming, verbal memory, working memory, attention, language, abstraction, and orientation	
	Craft Story Test	Short- and long-term verbal memory are measured with a related story	
	Rey Auditory Verbal Learning Test	Short- and long-term verbal memory are measured with a 15-word list	
	Benson Complex Figure	Short- and long-term visual memory and visuoconstruction are measured with a complex figure	
	Multilingual Naming Test	30-item test that measures naming ability	
	The Digit Span Test	Working memory and attention abilities are measured with series of numbers repeated in a forward and backward order	
	Verbal Fluency	Verbal phonological fluency is assessed by generating as many words as possible beginning with a specific letter (F, A, S, and M) in 1 min each	
	Category Fluency	Semantic fluency is assessed by generating as many words as possible from a specific category (animals, vegetables, and fruits) in 1 min each	
	Wisconsin Card Sorting Test-64	Executive function is measured by having the participant match cards according to certain rules (color, figure, and number of figures in card)	
	Boston Naming Test	15-item test that measures naming ability	
	Symbol Digit Modality Test	Speeded information processing is assessed by associating numbers with symbols as fast as possible in 90 seconds	
	Word Accentuation Test Chicago	Participants read a list of words and attempt to place the correct accentuation, measuring premorbid intelligence	
**Feasibility**			
	Teleneuropsychological Assessment Satisfaction Scale	Provides participants with an opportunity to give feedback on various aspects of the assessment, including satisfaction, the use of Zoom and REDCap, instructions, advantages, difficulties, recommendations, and overall time	

^a^TICS-M: modified Telephone Interview for Cognitive Status.

^b^NACC: National Alzheimer’s Coordinating Center.

^c^GDS: Geriatric Depression Scale.

^d^REDCap: Research Electronic Data Capture.

**Table 2:** Demographic and clinical characteristics of the study participants (n=67).

Variable			Participants
Age (y), mean (SD)			62.2 (7.6)
**Sex, n (%)**			
	Female	53 (79.1)	
**Race, n (%)**			
	Afro-Panamanian or Black	11 (16.4)	
	Asian	1 (1.5)	
	Indigenous people	2 (3.0)	
	White	31 (46.3)	
	Unknown	2 (3.0)	
	Other	20 (29.9)	
**Marital status, n (%)**			
	Partnered	44 (65.7)	
	Not partnered	23 (34.3)	
Education (y), mean (SD)			16.7 (1.9)
Years smoking, mean (SD)			15.3 (12.4)
Age at smoking cessation (years), mean (SD)			36.7
Diabetes (yes), n (%)			6 (9)
Hypertension (yes), n (%)			22 (32.8)
Hypercholesterolemia (yes), n (%)			19 (28.4)
Thyroid disease (yes), n (%)			13 (19.4)
Arthritis (yes), n (%)			9 (13.4)
Number of cardiovascular disease, mean (SD)			0.25 (0.8)
Number of cerebrovascular illnesses, mean (SD)			0.15 (0.5)
Number of medical conditions, mean (SD)			1.1 (1.0)
Number of sleep-related illnesses, mean (SD)			0.33 (0.6)
Number of psychiatric diseases, mean (SD)			0.33 (0.8)
Depression (yes), n (%)			11 (16.4)
Anxiety (yes), n (%)			7 (10.4)
Geriatric depression symptoms, mean (SD)			1.5 (1.8)
Functionality Index, mean (SD)			0.5 (0.8)

### Expected Outcomes for Assessing Feasibility

Feasibility will be assessed based on an analysis of the tools, funding, expertise, and resources needed to conduct neuropsychological assessments. Likewise, the Teleneuropsychological Assessment Satisfaction Scale will assess feasibility through the satisfaction of the sample with the teleneuropsychology assessment. The Teleneuropsychological Assessment Satisfaction Scale provides participants with an opportunity to give feedback on various aspects of the assessment, including satisfaction, the use of Zoom and REDCap, instructions, advantages, difficulties, recommendations, and overall time.

So far, the Teleneuropsychological Assessment Satisfaction Scale (Multimedia Appendix 1) has shown that most participants (39/67, 58%) were very satisfied with the teleneuropsychological assessment, while high levels of satisfaction were found with other elements of the assessment, such as the use of the Zoom platform (47/67, 70%), the evaluator (57/67, 85%), or the REDCap platform (33/67, 49%). All participants reported instructions were clear. Most participants (49/67, 73%) were satisfied with being able to do the evaluation from home. Two-thirds of participants (44/67, 66%) reported that they were able to use electronic equipment, such as computers, phones, or tablets. Most participants (37/67, 55%) would recommend a teleneuropsychological assessment to others.

Although most of the sample (53/67, 79%) reported no difficulties at all, main limitations that were stated by participants were internet connection (8/67, 12%), space in which the assessment took place (8/67, 12%), and electronic equipment used (5/67, 7%). Other barriers (12/67, 18%) included the long duration of the assessment, confusing wording in questionnaires, problems with sound, interruptions, difficulties with the assessment materials, problems with the Zoom platform, availability for the assessment, lack of light, and complaints regarding their own abilities.

### Expected Outcomes for Creating Normative Data

In line with the FLOAT study [63], percentile ranks for each test’s raw scores will be calculated from cumulative frequency distributions and then normalized to a distribution with a mean of 10 and an SD of 3 (ie, scaled scores). These normalized scores will then be entered into a stepwise regression, including sex (male and female), education, and different age groups. Predictors contributing <5% of additional variance in the normalized scores will be retained for further regression-based adjustments.

## Discussion

### Principal Findings

This paper describes the protocol of a pilot study designed to assess the feasibility of conducting teleneuropsychological cognitive assessments in adults aged ≥50 years. Teleneuropsychology is a technology-based practice that includes collecting patient data using multiple assessment modalities. So far, we have successfully assessed 67 participants with a complete teleneuropsychology evaluation. Our results indicate that our sample is comparable to those in other studies in terms of age [34,38], education level [80,81], and the distribution of men and women [40], characteristics that might contribute to the validity of our design in future analyses.

Moreover, feasibility could also be demonstrated through the successful use of various technological platforms in the assessments, including REDCap and Zoom. This approach to teleneuropsychological assessments has been recommended in other studies [5] and aligns with guidelines established before and during the COVID-19 pandemic [1,7]. To date, very few studies in Panama have used REDCap to capture and store clinical data; nevertheless, this is the first time it is used for neuropsychological assessments, making this an unprecedented study. REDCap has been widely used and recommended for teleneuropsychology studies across various regions worldwide [3,82], as data can only be accessed with a password and server administrator permissions, making the platform ideal for securely storing the collected information [83]. REDCap stands as a viable platform that could be part of the regular clinical practice, ensuring the security, conservation, and confidentiality of gathered data. Likewise, using Zoom as the platform for the assessment is a notable strength, as it has been effectively used in other similar studies [48,84].

In addition, current participant satisfaction has been assessed, revealing that most respondents (39/67, 58%) were highly satisfied with the remote assessment. This aligns with findings from previous studies on participant satisfaction [35,36,42,85]. Key positive aspects highlighted by participants included the use of electronic equipment, the convenience of conducting assessments from home, and the quality of interaction and communication with evaluators. These findings reinforce the strengths of teleneuropsychology identified in another research [41].

### Limitations

So far, participants reported difficulties primarily related to electronic equipment, internet connectivity, and the environment in which the assessment took place. These limitations are common challenges associated with teleneuropsychology [86,87], and as participants completed the assessment at home, these factors were beyond the assessor’s control. We expect other participants assessed to encounter technological difficulties. The use of REDCap presents both strengths and limitations. On the one hand, this platform ensures secure and confidential storage of test and questionnaire data, as it is only accessible to those connected to the research institution’s server. On the other hand, because the server hosting REDCap is owned by the institution, both the researcher and the participant must have access to the server to use the platform.

Other limitations include the use of convenience sampling for recruitment, which hinders the inclusion of a diverse sample. This resulted in participants with a high level of education and a medium to high socioeconomic level that are not representative of the population. We will actively aim to recruit participants that come from different backgrounds and diverse socioeconomic levels.

### Strengths

The study’s strengths include the innovative teleneuropsychology assessment format, which involved both the participant and assessor being at home, creating a teleneuropsychology “home-to-home” format, an approach that has been validated but not extensively studied [33,42,60,88,89]. This is of particular interest given that the use of teleneuropsychology home-to-home neuropsychological testing by mental health professionals has been reported in the Latin American region; however, no specific studies documenting this modality have been published to date [59], making this study one of the few to explore this method.

The application of the assessment battery enabled the collection of specific data from the Panamanian population, paving the way for the development of normative data for tests conducted remotely. This aims to address a significant limitation in the region, where standardized tests have not been adapted to the populations of these countries [90-93].

While some participants reported challenges related to connection issues, the assessment environment, and the equipment used, it is important to note that these difficulties were experienced by only a minority of the sample. In addition, specific issues related to the questionnaires emerged; participants noted that the wording and timing of the questions were not favorable. In contrast, the overall timing of the evaluation itself was well received by participants.

### Conclusions

Teleneuropsychology has gained ground in the field of psychology, offering new spaces and opportunities for professionals, patients, and participants. Ongoing research is essential to bring clarity to the clinical practice of those using these alternatives. Drawing on lessons learned before, during, and after the pandemic, many professionals have adopted models that combine elements of remote and traditional face-to-face care, giving rise to hybrid neuropsychology [94]. In Latin America, further research is essential, particularly studies aimed at developing instruments, models, and validations and establishing normative data for diverse populations in the region.

This study represents the first pilot investigation in Central America aimed at assessing the feasibility of a teleneuropsychology protocol and establishing normative data for a Panamanian sample. By administering cognitive assessments to individuals at low risk of developing dementia, we aim to generate data that can be compared with Hispanic samples from the United States, contributing to a broader understanding of cognitive performance across diverse populations. In addition, evaluating participant satisfaction with the teleneuropsychological evaluation process provides valuable insights into its acceptability and usability. Furthermore, analyzing performance differences across gender and age groups will help identify potential demographic influences on test outcomes. Ultimately, this research will contribute to the validation of remote cognitive assessments in Latin America, supporting their use as reliable and accessible tools for neuropsychological evaluation.

We recommend expanding the sample to establish normative data for older adults in Panama. This will require strengthening participant recruitment efforts across diverse regions of the country to maximize the accessibility and advantages of teleneuropsychology. Likewise, this study can be replicated to use teleneuropsychology assessments with other age ranges and populations, such as children, adolescents, and middle-aged adults. In addition, it is imperative to conduct a similar study with individuals with mild cognitive impairment to explore applications in neurodegenerative conditions such as Alzheimer and Parkinson disease. Finally, in studies such as ours, we recommend incorporating medical records in future studies to confirm participants’ health status, aligning with best practices used in research worldwide.

## Appendix

Multimedia Appendix 1 Satisfaction with the teleneuropsychological assessment.

## Abbreviations

ADRD: Alzheimer disease and related dementias
FLOAT: Development and Validation of Teleneuropsychology Normative Data for Older Adults in Florida
GDS: Geriatric Depression Scale
NACC: National Alzheimer’s Coordinating Center
REDCap: Research Electronic Data Capture
TICS-M: modified Telephone Interview for Cognitive Status

## Data Availability

Results from this study will be available upon completion and publication of the primary findings, upon reasonable request and with appropriate ethics approvals and data use agreements.

## References

[1] Bilder RM, Postal KS, Barisa M, Aase DM, Cullum CM, Gillaspy SR, Harder L, Kanter G, Lanca M, Lechuga DM, Morgan JM, Most R, Puente AE, Salinas CM, Woodhouse J (2020). Inter organizational practice committee recommendations/guidance for teleneuropsychology in response to the COVID-19 pandemic. Arch Clin Neuropsychol.

[2] Parsons T, Duffield T (2020). Paradigm shift toward digital neuropsychology and high-dimensional neuropsychological assessments: review. J Med Internet Res.

[3] Crivelli L, Quiroz YT, Calandri IL, Martin ME, Velilla LM, Cusicanqui MI, Yglesias FC, Llibre-Rodríguez JJ, Armele M, Román F, Barceló E, Dechent C, Carello MA, Olavarría L, Yassuda MS, Custodio N, Dansilio S, Sosa AL, Acosta DM, Brucki SM, Caramelli P, Slachevsky A, Nitrini R, Carrillo MC, Allegri RF (2022). working group recommendations for the practice of teleneuropsychology in Latin America. Arch Clin Neuropsychol.

[4] Tele-neuropsychology guidelines. Inter Organizational Practice Committee.

[5] Kitaigorodsky M, Loewenstein D, Curiel Cid R, Crocco E, Gorman K, González-Jiménez C (2021). A teleneuropsychology protocol for the cognitive assessment of older adults during COVID-19. Front Psychol.

[6] Joint Task Force for the Development of Telepsychology Guidelines for Psychologists (2013). Guidelines for the practice of telepsychology. Am Psychol.

[7] Grosch MC, Gottlieb MC, Cullum CM (2011). Initial practice recommendations for teleneuropsychology. Clin Neuropsychol.

[8] Welsh K, Breitner J, Magruder-Habib K (1993). Detection of dementia in the elderly using telephone screening of cognitive status. Neuropsychiatry Neuropsychol Behav Neurol.

[9] Marcus AC, Crane LA (1986). Telephone surveys in public health research. Med Care.

[10] Ball C, Puffett A (1998). The assessment of cognitive function in the elderly using videoconferencing. J Telemed Telecare.

[11] Montani C, Billaud N, Couturier P, Fluchaire I, Lemaire R, Malterre C, Lauvernay N, Piquard J F, Frossard M, Franco A (1996). "Telepsychometry": a remote psychometry consultation in clinical gerontology: preliminary study. Telemed J.

[12] Brandt J (1991). The hopkins verbal learning test: development of a new memory test with six equivalent forms. Clin Neuropsychol.

[13] Roccaforte WH, Burke WJ, Bayer BL, Wengel SP (1992). Validation of a telephone version of the mini-mental state examination. J Am Geriatr Soc.

[14] Lanska DJ, Schmitt FA, Stewart JM, Howe JN (1993). Telephone-assessed mental state. Dementia.

[15] Roccaforte WH, Burke WJ, Bayer BL, Wengel SP (1994). Reliability and validity of the Short Portable Mental Status Questionnaire administered by telephone. J Geriatr Psychiatry Neurol.

[16] Kawas C, Karagiozis H, Resau L, Corrada M, Brookmeyer R (1995). Reliability of the blessed telephone information-memory-concentration test. J Geriatr Psychiatry Neurol.

[17] Gatz M, Reynolds C, Nikolic J, Lowe B, Karel M, Pedersen N (1995). An empirical test of telephone screening to identify potential dementia cases. Int Psychogeriatr.

[18] Go RC, Duke LW, Harrell LE, Cody H, Bassett SS, Folstein MF, Albert MS, Foster JL, Sharrow NA, Blacker D (1997). Development and validation of a structured telephone interview for dementia assessment (STIDA): the NIMH genetics initiative. J Geriatr Psychiatry Neurol.

[19] Debanne SM, Patterson MB, Dick R, Riedel TM, Schnell A, Rowland DY (1997). Validation of a telephone cognitive assessment battery. J Am Geriatr Soc.

[20] Buschke H, Kuslansky G, Katz M, Stewart W, Sliwinski M, Eckholdt H, Lipton R (1999). Screening for dementia with the memory impairment screen. Neurology.

[21] Norton MC, Tschanz JA, Fan X, Plassman BL, Welsh-Bohmer KA, West N, Wyse BW, Breitner JC (1999). Telephone adaptation of the modified mini-mental state exam (3MS). The cache county study. Neuropsychiatry Neuropsychol Behav Neurol.

[22] Rabin LA, Saykin AJ, Wishart HA, Nutter-Upham KE, Flashman LA, Pare N, Santulli RB (2007). The memory and aging telephone screen: development and preliminary validation. Alzheimers Dement.

[23] Beeri MS, Werner P, Davidson M, Schmidler J, Silverman J (2003). Validation of the modified telephone interview for cognitive status (TICS-m) in Hebrew. Int J Geriatr Psychiatry.

[24] Kliegel M, Martin M, Jäger T (2007). Development and validation of the cognitive telephone screening instrument (COGTEL) for the assessment of cognitive function across adulthood. J Psychol.

[25] Castanho TC, Amorim L, Zihl J, Palha JA, Sousa N, Santos NC (2014). Telephone-based screening tools for mild cognitive impairment and dementia in aging studies: a review of validated instruments. Front Aging Neurosci.

[26] McKenna P, Warrington E, Grant I, Adams KM (2009). The analytical approach to neuropsychological assessment. Neuropsychological Assessment of Neuropsychiatric Disorders.

[27] Mitsis EM, Jacobs D, Luo X, Andrews H, Andrews K, Sano M (2010). Evaluating cognition in an elderly cohort via telephone assessment. Int J Geriatr Psychiatry.

[28] Conwell Y, Simning A, Driffill N, Xia Y, Tu X, Messing SP, Oslin D (2018). Validation of telephone-based behavioral assessments in aging services clients. Int Psychogeriatr.

[29] Barak A, Hen L, Boniel-Nissim M, Shapira N (2008). A comprehensive review and a meta-analysis of the effectiveness of internet-based psychotherapeutic interventions. J Technol Hum Serv.

[30] Backhaus A, Agha Z, Maglione ML, Repp A, Ross B, Zuest D, Rice-Thorp NM, Lohr J, Thorp SR (2012). Videoconferencing psychotherapy: a systematic review. Psychol Serv.

[31] Ceslis A, Mackenzie L, Robinson GA (2022). Implementation of a hybrid teleneuropsychology method to assess middle aged and older adults during the COVID-19 pandemic. Arch Clin Neuropsychol.

[32] Brearly TW, Shura RD, Martindale SL, Lazowski RA, Luxton DD, Shenal BV, Rowland JA (2017). Neuropsychological test administration by videoconference: a systematic review and meta-analysis. Neuropsychol Rev.

[33] Alegret M, Espinosa A, Ortega G, Pérez-Cordón A, Sanabria Á, Hernández I, Marquié M, Rosende-Roca M, Mauleón A, Abdelnour C, Vargas L, de Antonio EE, López-Cuevas R, Tartari JP, Alarcón-Martín E, Tárraga L, Ruiz A, Boada M, Valero S (2021). From face-to-face to home-to-home: validity of a teleneuropsychological battery. J Alzheimers Dis.

[34] Chapman JE, Gardner B, Ponsford J, Cadilhac DA, Stolwyk RJ (2020). Comparing performance across in-person and videoconference-based administrations of common neuropsychological measures in community-based survivors of stroke. J Int Neuropsychol Soc.

[35] Parikh M, Grosch MC, Graham LL, Hynan LS, Weiner M, Shore JH, Cullum CM (2013). Consumer acceptability of brief videoconference-based neuropsychological assessment in older individuals with and without cognitive impairment. Clin Neuropsychol.

[36] Appleman ER, O'Connor MK, Boucher SJ, Rostami R, Sullivan SK, Migliorini R, Kraft M (2021). Teleneuropsychology clinic development and patient satisfaction. Clin Neuropsychol.

[37] Vahia IV, Ng B, Camacho A, Cardenas V, Cherner M, Depp CA, Palmer BW, Jeste DV, Agha Z (2015). Telepsychiatry for neurocognitive testing in older rural Latino adults. Am J Geriatr Psychiatry.

[38] Wadsworth HE, Galusha-Glasscock JM, Womack KB, Quiceno M, Weiner MF, Hynan LS, Shore J, Cullum CM (2016). Remote neuropsychological assessment in rural American Indians with and without cognitive impairment. Arch Clin Neuropsychol.

[39] Hammers DB, Stolwyk R, Harder L, Cullum CM (2020). A survey of international clinical teleneuropsychology service provision prior to and in the context of COVID-19. Clin Neuropsychol.

[40] Marra DE, Hamlet KM, Bauer RM, Bowers D (2020). Validity of teleneuropsychology for older adults in response to COVID-19: a systematic and critical review. Clin Neuropsychol.

[41] Perez P, Ramos D, Arango JC (2021). Teleneuropsicología en países de habla hispana: Una mirada crítica al uso de Tecnologías de Información y Comunicación en la evaluación neuropsicológica. Rev Iberoam Neuropsic.

[42] Parsons MW, Gardner MM, Sherman JC, Pasquariello K, Grieco JA, Kay CD, Pollak LE, Morgan AK, Carlson-Emerton B, Seligsohn K, Davidsdottir S, Pulsifer MB, Zarrella GV, Burstein SM, Mancuso SM (2021). Feasibility and acceptance of direct-to-home tele-neuropsychology services during the COVID-19 pandemic. J Int Neuropsychol Soc.

[43] Rochette AD, Rahman-Filipiak A, Spencer RJ, Marshall D, Stelmokas JE (2022). Teleneuropsychology practice survey during COVID-19 within the United States. Appl Neuropsychol Adult.

[44] Arias F, Safi DE, Miranda M, Carrión CI, Diaz Santos AL, Armendariz V, Jose IE, Vuong KD, Suarez P, Strutt AM, STAR Consortium (2020). Teleneuropsychology for monolingual and bilingual Spanish-speaking adults in the time of COVID-19: rationale, professional considerations, and resources. Arch Clin Neuropsychol.

[45] Sperling SA, Acheson SK, Fox-Fuller J, Colvin MK, Harder L, Cullum CM, Randolph JJ, Carter KR, Espe-Pfeifer P, Lacritz LH, Arnett PA, Gillaspy SR (2024). Tele-neuropsychology: from science to policy to practice. Arch Clin Neuropsychol.

[46] Becerra M El progreso inconcluso de inclusión digital en América Latina. Fundación Telefónica España.

[47] Schade Y. N, Medina J. F, Ramírez-Vielma R, Sanchez-Cabaco A, De La Torre L. L (2022). Detección temprana de Deterioro Cognitivo Leve en personas mayores durante la pandemia: protocolo cribado online. Rev Chil Neuro Psiquiatr.

[48] Fox-Fuller JT, Ngo J, Pluim CF, Kaplan RI, Kim D, Anzai JAU, Yucebas D, Briggs SM, Aduen PA, Cronin-Golomb A, Quiroz YT (2022). Initial investigation of test-retest reliability of home-to-home teleneuropsychological assessment in healthy, English-speaking adults. Clin Neuropsychol.

[49] Montaña Luque S, Lopera Vásquez J, Martínez Morales D, Carvajal Castrillón J, Galeano Toro LM, Rueda Nobmann MT, García-Giraldo ?M, Garzón Giraldo LD, Arias Ramírez Y, De La Torre Salazar D, Carmona Castaño LF, Clara Jaramillo J, Pérez Restrepo P, Castrillón Taba MM, Uribe Lopera A, Yibirín Peinado C, Moreno Carrillo C, Méndez Barrera L, Torres Bustamante N, Vélez Córdoba A, Madrid Loaiza EA, Pizano Cardona L, Dávila Plata D, Vergara Góez E, Botero Guerra N, Arboleda Ramirez A (2021). Teleneuropsicología: experiencia del Instituto Neurológico de Colombia durante confinamiento obligatorio por covid-19, año 2020. J Contin Publ.

[50] Messler AC, Kane KD, Serrano Y (2025). Tele-neuropsychology in culturally and linguistically diverse populations within the U.S. and U.S. territories: a scoping review. Clin Neuropsychol.

[51] Sánchez Cabaco A, De La Torre L, Alvarez Núñez DN, Mejía Ramírez MA, Wöbbeking Sánchez M (2023). Tele neuropsychological exploratory assessment of indicators of mild cognitive impairment and autonomy level in Mexican population over 60 years old. PEC Innov.

[52] de Araújo Silva JD, Maranhão DC, Beltrão NB, Farah BQ, Damasceno VD, Cavalcante BR, Pirauá AL (2023). Videoconference assessment of functional and cognitive measures in Brazilian older adults: a reliability and feasibility study. Geriatr Gerontol Aging.

[53] Messler AC, Hargrave DD, Trittschuh EH, Sordahl J (2025). National survey of telehealth neuropsychology practices: current attitudes, practices, and relevance of tele-neuropsychology three years after the onset of Covid-19. Clin Neuropsychol.

[54] Montemurro S, Mondini S, Pucci V, Durante G, Riccardi A, Maffezzini S, Scialpi G, Signorini M, Arcara G (2023). Tele-Global Examination of Mental State (Tele-GEMS): an open tool for the remote neuropsychological screening. Neurol Sci.

[55] Rivella C, Ruffini C, Bombonato C, Capodieci A, Frascari A, Marzocchi GM, Mingozzi A, Pecini C, Traverso L, Usai MC, Viterbori P (2023). TeleFE: a new tool for the tele-assessment of executive functions in children. Appl Sci.

[56] Wadsworth HE, Dhima K, Womack KB, Hart J, Weiner MF, Hynan LS, Cullum CM (2018). Validity of teleneuropsychological assessment in older patients with cognitive disorders. Arch Clin Neuropsychol.

[57] Hunter MB, Jenkins N, Dolan C, Pullen H, Ritchie C, Muniz-Terrera G (2021). Reliability of telephone and videoconference methods of cognitive assessment in older adults with and without dementia. J Alzheimers Dis.

[58] Arruabarrena MM, Martin ME, Calandri IL, Corvalán N, Helou M, Martínez C, Crivelli L (2022). Teleneuropsychological assessment in South America: a perspective from patients and neuropsychologists. J Appl Cogn Neurosci (Barranquilla).

[59] Seubert-Ravelo AN, Serrano-Juárez CA, Cabañas-Tinajero JÁ, González-Gutiérrez FA, Moreno-Villagómez J, Prieto-Corona B, Reyes-Méndez C, Téllez-Rodríguez M, Yáñez-Téllez MG (2023). Teleneuropsychology during the COVID-19 pandemic in Mexico: the perspective from a middle-income country. J Clin Exp Neuropsychol.

[60] Moreau J, Pollock B, Harrison AG (2023). In-person and in-home teleneuropsychological assessments with youth with neurodevelopmental disorders: what’s the difference?. Can J Sch Psychol.

[61] Serrano-Juárez CA, Reyes-Méndez C, Prieto-Corona B, Seubert-Ravelo AN, Moreno-Villagómez J, Cabañas-Tinajero JÁ, Yáñez-Téllez MG, Quezada-Torres RA, Téllez-Rodríguez M, Barrera-Rodríguez B, Soto-Jiménez MP, González-Gutiérrez FA, Castillo-Tejeda E (2023). A systematic review and a Latin American clinical model for teleneuropsychological assessment. Arch Clin Neuropsychol.

[62] Santos Mejía L, Oviedo D, Pérez-Lao A, Britton GB (2023). Review of the use of teleneuropsychology in Latin American populations. Investigación Y Pensamiento Crítico.

[63] Perez-Lao A, Ying G, Mitova E, Morales A, Marra D, Arias F, Levy S, Smith G (2025). Study protocol using informatics to identify and recruit a cohort of older adults in Florida to develop teleneuropsychological norms. BMJ Open.

[64] Harris PA, Taylor R, Thielke R, Payne J, Gonzalez N, Conde JG (2009). Research electronic data capture (REDCap)--a metadata-driven methodology and workflow process for providing translational research informatics support. J Biomed Inform.

[65] Cook SE, Marsiske M, McCoy KJ (2009). The use of the modified Telephone Interview for Cognitive Status (TICS-M) in the detection of amnestic mild cognitive impairment. J Geriatr Psychiatry Neurol.

[66] (2006). NACC uniform data set instructions for the neuropsychological battery. NACC.

[67] Kurlowicz L (2000). The Geriatric Depression Scale (GDS). Insight.

[68] Nasreddine ZS, Phillips NA, Bédirian V, Charbonneau S, Whitehead V, Collin I, Cummings JL, Chertkow H (2005). The Montreal Cognitive Assessment, MoCA: a brief screening tool for mild cognitive impairment. J Am Geriatr Soc.

[69] Howard RS, Goldberg TE, Luo J, Munoz C, Schneider LS (2023). Reliability of the NACC Telephone-administered Neuropsychological Battery (T-cog) and Montreal Cognitive Assessment for participants in the USC ADRC. Alzheimers Dement (Amst).

[70] Rey A (1958). L'examen clinique en psychologie.

[71] Osterrieth PA (1944). Le test de copie d'une figure complexe; contribution à l'étude de la perception et de la mémoire Test of copying a complex figure; contribution to the study of perception and memory. Arch Psychol.

[72] Gollan TH, Weissberger GH, Runnqvist E, Montoya RI, Cera CM (2012). Self-ratings of spoken language dominance: a Multi-Lingual Naming Test (MINT) and preliminary norms for young and aging Spanish English bilinguals. Biling (Camb Engl).

[73] Wechsler D Wechsler adult intelligence scale--3rd edition (WAIS-III). PsycTest.

[74] Lezak M, Howieson D, Bigler E, Tranel D (2012). Neuropsychological Assessment.

[75] Grant DA, Berg E (1948). A behavioral analysis of degree of reinforcement and ease of shifting to new responses in a Weigl-type card-sorting problem. J Exp Psychol.

[76] Abraham E, Axelrod BN, Ricker JH (1996). Application of the oral trail making test to a mixed clinical sample. Arch Clin Neuropsychol.

[77] Kaplan E, Goodglass H (1983). Boston Naming Test.

[78] Benedict RH, DeLuca J, Phillips G, LaRocca N, Hudson LD, Rudick R, Multiple Sclerosis Outcome Assessments Consortium (2017). Validity of the symbol digit modalities test as a cognition performance outcome measure for multiple sclerosis. Mult Scler.

[79] Krueger KR, Lam CS, Wilson RS (2006). The word accentuation test - Chicago. J Clin Exp Neuropsychol.

[80] Cullum CM, Weiner MF, Gehrmann HR, Hynan LS (2006). Feasibility of telecognitive assessment in dementia. Assessment.

[81] Munro Cullum C, Hynan LS, Grosch M, Parikh M, Weiner MF (2014). Teleneuropsychology: evidence for video teleconference-based neuropsychological assessment. J Int Neuropsychol Soc.

[82] Tailby C, Collins AJ, Vaughan DN, Abbott DF, O'Shea M, Helmstaedter C, Jackson GD (2020). Teleneuropsychology in the time of COVID-19: the experience of The Australian Epilepsy Project. Seizure.

[83] Home page. REDCap.

[84] Chapman JE, Cadilhac DA, Gardner B, Ponsford J, Bhalla R, Stolwyk RJ (2019). Comparing face-to-face and videoconference completion of the Montreal Cognitive Assessment (MoCA) in community-based survivors of stroke. J Telemed Telecare.

[85] Lacritz LH, Carlew AR, Livingstone J, Bailey KC, Parker A, Diaz A (2020). Patient satisfaction with telephone neuropsychological assessment. Arch Clin Neuropsychol.

[86] Gardner M, Aslanzadeh F, Zarrella GV, Braun SE, Loughan AR, Parsons MW (2021). Cancer, cognition, and COVID: delivering direct-to-home teleneuropsychology services to neuro-oncology patients. Neurooncol Pract.

[87] Tsiakiri A, Koutzmpi V, Megagianni S, Toumaian M, Geronikola N, Despoti A, Kanellopoulou S, Arampatzi X, Margioti E, Davila A, Zoi P, Kalligerou F, Liozidou A, Tsapanou A, Sakka P (2024). Remote neuropsychological evaluation of older adults. Appl Neuropsychol Adult.

[88] Hantke NC, Gould C (2020). Examining older adult cognitive status in the time of COVID-19. J Am Geriatr Soc.

[89] Parks AC, Davis J, Spresser CD, Stroescu I, Ecklund-Johnson E (2021). Validity of in-home teleneuropsychological testing in the wake of COVID-19. Arch Clin Neuropsychol.

[90] Ardila A, Rosselli M, Puente A (1994). Neuropsychological Evaluation of the Spanish Speaker.

[91] García de la Cadena C, Henríquez J, Sequeira E, Cortés OA, De OR, Judd T (2009). La Neuropsicología en América Central. Rev Neuropsic Neuropsiquiatr.

[92] Pérez-Parra JE, Puerta-Lopera IC, Dussán-Lubert C, Montoya-Londoño DM, Landínez-Martínez D (2022). Validación y estandarización de pruebas neuropsicológicas para la evaluación de funciones ejecutivas en población universitaria. Cuad Hispanoam Psicol.

[93] Puerta Lopera IC, Dussán Lubert C, Montoya Londoño DM, Landínez Martínez D (2019). Standardization of a protocol of neuropsychological tests for the assessment of attention in college students. CES Psico.

[94] Singh S, Germine L (2021). Technology meets tradition: a hybrid model for implementing digital tools in neuropsychology. Int Rev Psychiatry.

