# Supplementary material for: A Teleneuropsychological Battery for Assessing Older Panamanian Adults: Protocol for a Cross-Sectional Pilot Feasibility Study
Source: JMIR Res Protoc. 2025 Aug 28;14:e68520. doi: 10.2196/68520 (PMC12428167; doi:10.2196/68520)
Supplement: Multimedia Appendix 1 [file resprot_v14i1e68520_app1.docx]

| **Appendix 1.** *Satisfaction with the teleneuropsychological assessment* | |
| --- | --- |
| **Select your degree of satisfaction with the teleneuropsychological assessment**  (*n = 65)* | *n (%) / M (DE)* |
| Very dissatisfied | 1 (1.5%) |
| Dissatisfied | 0 (0.0%) |
| Neutral | 3 (4.6%) |
| Satisfied | 22 (33.8%) |
| Very satisfied | 39 (60.0%) |
| **Were you satisfied with the use of Zoom?** |  |
| Very dissatisfied | 0 (0.0%) |
| Dissatisfied | 0 (0.0%) |
| Neutral | 2 (3.1%) |
| Satisfied | 16 (24.6%) |
| Very satisfied | 47 (63.5%) |
| **Were you satisfied with the evaluator´s treatment?** |  |
| Very dissatisfied | 0 (0.0%) |
| Dissatisfied | 1 (1.5%) |
| Neutral | 0 (0.0%) |
| Satisfied | 7 (10.8%) |
| Very satisfied | 57 (87.7%) |
| **Were you satisfied with the REDCap Platform?** |  |
| Very dissatisfied | 1 (1.4%) |
| Dissatisfied | 0 (0.0%) |
| Neutral | 5 (7.7%) |
| Satisfied | 26 (40.0%) |
| Very satisfied | 33 (50.8%) |
| **I was able to easily understand the instructions given to me to complete the tests** |  |
| Agree | 26 (40.0%) |
| Strongly agree | 39 (60.0%) |
| **Aspects of the assessment that you liked** |  |
| Being able to do it from home or a place near home | 49 (75.4%) |
| Being able to use computers/cellphone/tablet to answer the tests | 44 (67.7%) |
| The communication with the evaluators | 41 (63.1%) |
| The attention given to me by the evaluators | 46 (70.8%) |
| Other (Feeling competent, helping the research, wished to know more, etc) | 7 (10.8%) |
| **Aspects of the assessment that you had difficulties with** |  |
| With internet connection | 8 (12.3%) |
| With the space where I was during the assessment | 8 (12.3%) |
| With the computer/cellphone/tablet that I used during the assessment | 5 (7.7%) |
| With the evaluators | 1 (1.5%) |
| Other | 12 (18.5%) |
| I had no difficulties | 53 (81.5%) |
| **Adequate time for the assessment** |  |
| Yes | 59 (90.8%) |
| No | 6 (9.2%) |
| **Reasons why time wasn’t adequate** |  |
| I recommend dividing the assessment | 1 (1.5%) |
| Application of the tests took too much time | 1 (1.5%) |
| Assessment time is too long | 4 (6.2%) |
| **What would you change of the assessment?** |  |
| The initial contact with the investigators (when I was called to participate) | 2 (3.1%) |
| REDCap Platform | 1 (1.5%) |
| The questionnaires used | 10 (15.4%) |
| The test used | 1 (1.5%) |
| The time used for the assessment | 10 (15.4%) |
| I wouldn´t change anything | 41 (63.1%) |
| **Would recommend a TNP evaluation** |  |
| Strongly disagree | 0 (0.0%) |
| Disagree | 1 (1.5%) |
| Neutral | 3 (4.6%) |
| Slightly agree | 3 (4.6%) |
| Agree | 21 (32.3%) |
| Strongly agree | 37 (56.9%) |
